# Protein Misfolding and Aggregation as a Mechanistic Link Between Chronic Pain and Neurodegenerative Diseases

**DOI:** 10.3390/cimb47040259

**Published:** 2025-04-08

**Authors:** Nebojsa Brezic, Strahinja Gligorevic, Aleksandar Sic, Nebojsa Nick Knezevic

**Affiliations:** 1 Department of Anesthesiology, Advocate Illinois Masonic Medical Center, Chicago, IL 60657, USA; nebojsabrezic@gmail.com (N.B.); strahinja.gligorevic99@gmail.com (S.G.); aca.smed01@gmail.com (A.S.); 2 Faculty of Medicine, University of Belgrade, 11000 Belgrade, Serbia; 3 Department of Anesthesiology, University of Illinois, Chicago, IL 60612, USA; 4 Department of Surgery, University of Illinois, Chicago, IL 60612, USA

**Keywords:** chronic pain, neurodegenerative diseases, protein misfolding, protein aggregation, unfolded protein response, endoplasmic reticulum stress, neuroinflammation

## Abstract

Chronic pain, defined by persistent pain beyond normal healing time, is a pervasive and debilitating condition affecting up to 30–50% of adults globally. In parallel, neurodegenerative diseases (NDs) such as Alzheimer’s disease (AD), Parkinson’s disease (PD), and amyotrophic lateral sclerosis (ALS) are characterized by progressive neuronal loss and cognitive or motor decline, often underpinned by pathological protein misfolding and aggregation. Emerging evidence suggests a potential mechanistic link between chronic pain and NDs, with persistent pain contributing to neuroinflammatory states and protein homeostasis disturbances that mirror processes in neurodegeneration. This review explores the hypothesis that protein misfolding and aggregation serve as a mechanistic bridge between chronic pain and neurodegeneration. We systematically examine molecular pathways of protein misfolding, proteostasis dysfunction in chronic pain, and shared neuroimmune mechanisms, highlighting prion-like propagation of misfolded proteins, chronic neuroinflammation, and oxidative stress as common denominators. We further discuss evidence from experimental models and clinical studies linking chronic pain to accelerated neurodegenerative pathology—including tau accumulation, amyloid dysregulation, and microglial activation—and consider how these insights open avenues for novel therapeutics. Targeting protein aggregation, enhancing chaperone function, modulating the unfolded protein response (UPR), and attenuating glial activation are explored as potential strategies to mitigate chronic pain and possibly slow neurodegeneration. Understanding this intersection not only elucidates chronic pain’s role in cognitive decline but also suggests that interventions addressing proteostasis and inflammation could yield dual benefits in pain management and neurodegenerative disease modification.

## 1. Introduction

Chronic pain and neurodegenerative diseases (NDs) are two seemingly distinct entities that increasingly converge in clinical and molecular research. Chronic pain is traditionally defined as pain persisting for over three months, exceeding the expected duration of tissue healing [1]. It afflicts a substantial portion of the global population—estimates range from 20% to as high as 63%, depending on criteria and populations studied [2,3]. Chronic pain presents in multiple forms and categories, including neuropathic, nociceptive, musculoskeletal, inflammatory, psychogenic, and mechanical types [4]. Low back pain is a leading contributor and exemplifies the socio-economic burden of chronic pain, with hundreds of millions affected worldwide [5]. Beyond physical suffering, chronic pain exacts a heavy psychological toll, often co-occurring with depression, anxiety, and decreased quality of life [6]. Management remains challenging; effective care typically requires multimodal strategies combining pharmacological treatments and non-pharmacological therapies [7].

Neurodegenerative diseases, on the other hand, are chronic disorders characterized by progressive loss of neurons and synaptic function. Common NDs include Alzheimer’s disease (AD), Parkinson’s disease (PD), and amyotrophic lateral sclerosis (ALS), among others. Despite differences in their clinical presentations, these conditions share overlapping pathogenic mechanisms [8]. Key among these are abnormal protein misfolding and aggregation, mitochondrial dysfunction, oxidative stress, and chronic neuroinflammation [8]. In AD, for example, misfolded β-amyloid (Aβ) peptides accumulate into extracellular plaques, and tau protein aggregates into intracellular neurofibrillary tangles, disrupting neuronal communication and triggering synaptic loss [9]. PD pathology is highlighted by intracellular α-synuclein inclusions (Lewy bodies) in dopaminergic neurons, accompanied by mitochondrial impairments and autophagy dysfunction [10]. In ALS, motor neurons accumulate toxic protein inclusions, including TAR DNA-binding protein 43 (TDP-43), superoxide dismutase 1 (SOD1), and fused in sarcoma (FUS), alongside glutamate excitotoxicity and neuroinflammation [11]. Notably, protein misfolding in these diseases can propagate in a prion-like fashion, seeding further aggregation and spreading pathology through neural circuits [12]. These overlapping pathways have prompted a view of NDs as “proteinopathies” rooted in failures of proteostasis—the cellular systems that maintain protein folding, trafficking, and degradation.

Traditionally, chronic pain and NDs have been studied in isolation. However, accumulating evidence suggests significant overlap and interplay between them [13,14]. Epidemiologically, chronic pain in mid- to late-life is associated with accelerated cognitive decline and increased risk of dementia [15]. A 2017 longitudinal cohort study reported that persistent pain was linked to faster memory decline and a higher incidence of Alzheimer’s and related dementias [16]. More recent analyses extend these findings. A 2020 systematic review posited that chronic pain and common pain conditions like arthritis may contribute to subsequent cognitive impairment and dementia, perhaps via sustained neuroinflammation and stress responses [15]. Biological data support these associations. In rodent models, induction of chronic pain (e.g., via sciatic nerve ligation or inflammatory injury) led to tau hyperphosphorylation and accumulation in the hippocampus, along with memory deficits [17]. In humans, older adults with chronic pain show higher cerebrospinal fluid (CSF) and plasma markers of neurodegeneration: elevated tau and neurofilament light chain (NfL) [18]. Neuroimaging corroborates these findings; for instance, individuals with chronic low back pain have increased levels of translocator protein (TSPO), a marker of glial activation, in several brain regions upon TSPO-PET imaging, suggesting a potential pain-protective and anti-inflammatory role [19].

A central question arises from these observations—could protein misfolding and aggregation serve as a mechanistic bridge between chronic pain and neurodegeneration? This hypothesis posits that the prolonged neuronal activity, stress, and inflammation inherent in chronic pain might induce proteostasis disturbances—such as endoplasmic reticulum stress, impaired protein degradation, and misfolded protein accumulation—that parallel early events in NDs. Chronic pain’s neuroinflammatory milieu (e.g., elevated cytokines like tumor necrosis factor (TNF), IL-6, IL-1β) may exacerbate oxidative and proteotoxic stress on neurons and glia, promoting pathological aggregation of proteins that in turn seeds neurodegenerative changes. Conversely, individuals with prodromal or early neurodegenerative changes might experience heightened pain sensitivity or chronic pain syndromes due to shared pathways of neural sensitization and immune activation—an idea supported by reports of increased pain in PD and AD patients beyond what comorbidities alone would explain [13,20]. Thus, protein misfolding and aggregation emerge as compelling candidates for this “two-way” interaction: they are not only a cause of neurodegeneration but may also be a consequence of chronic neural stress in pain states, potentially feeding forward into a vicious cycle of neurodegeneration.

In this review, we aim to (1) delineate the molecular mechanisms of protein misfolding and aggregation, particularly as they relate to neuronal health and disease; (2) examine evidence for proteostasis disturbances and misfolded protein accumulation in chronic pain conditions, identifying mechanistic insights from both animal models and clinical studies; (3) explore the common pathways linking chronic pain and neurodegeneration, including neuroimmune interactions, glial cell activation, and prion-like spread of protein aggregates; and (4) discuss therapeutic implications of this nexus, highlighting strategies targeting protein misfolding, neuroinflammation, and related pathways that might benefit patients suffering from chronic pain, neurodegenerative diseases, or both. By integrating findings across pain research and neurodegeneration, we hope to clarify whether chronic pain merely coexists with NDs or actively contributes to their pathogenesis via shared protein homeostasis pathways. Such insight could reshape our approach to managing chronic pain in populations at risk for NDs and encourage early interventions that preserve proteostasis and cognitive function.

## 2. Materials and Methods

A comprehensive literature search was performed across three major bibliographic databases: PubMed (including MEDLINE), Scopus, and Web of Science. Additional searches were conducted in Google Scholar to identify any relevant articles not indexed in the primary databases. No time restrictions were applied in the literature search to ensure the inclusion of all relevant studies on the topic. We combined keywords and medical subject headings (MeSH) terms reflecting the core concepts of this review. Key search terms included “chronic pain”, “neurodegeneration”, “neurodegenerative diseases”, “protein misfolding”, “protein aggregation”, “proteostasis”, “unfolded protein response (UPR)”, “endoplasmic reticulum stress”, “prion-like propagation”, “amyloid”, “tau”, “alpha-synuclein”, “TDP-43”, “microglia”, “neuroinflammation”, and “glial activation”. These terms were used in various Boolean combinations. While no language restrictions were imposed, the review primarily focused on literature published in English. We included studies of all designs to ensure a wide net was cast. Reference lists of pertinent articles and prior reviews were hand-searched to identify additional relevant studies.

We included articles that met the following criteria: (a) peer-reviewed publications presenting primary data or significant secondary analysis on chronic pain (any etiology) with measured outcomes relevant to protein misfolding, aggregation, or proteostasis (e.g., levels of misfolded proteins, chaperone expression, proteasome or autophagy activity, UPR markers) or examining neurodegenerative disease pathology in the context of chronic pain; (b) studies on neurodegenerative diseases where chronic pain or nociceptive phenomena were assessed alongside protein aggregation pathology or related molecular pathways; (c) mechanistic studies exploring how chronic pain conditions affect molecular pathways of protein folding, quality control, or degradation in the nervous system; and (d) therapeutic studies if they specifically targeted protein misfolding/aggregation pathways in models of chronic pain or in patients with pain. We excluded (a) non-peer-reviewed material to maintain scientific rigor; (b) studies focusing solely on acute pain or perioperative pain without a chronic component; (c) studies that discussed protein misfolding or neurodegeneration without any connection to pain; and (d) case reports or small case series unless they provided unique mechanistic insight. Where multiple similar studies existed, we favored inclusion of systematic reviews or meta-analyses for summary, unless newer primary studies provided updated data. All figures were created using Microsoft PowerPoint for Mac Version 16.83 (24031102) (Microsoft Corporation, Redmond, WA, USA) for schematic representation and visualization of proposed mechanisms.

## 3. Protein Misfolding and Aggregation: Molecular Mechanisms

Proteins must fold into precise three-dimensional structures to function correctly. “Protein misfolding” refers to the process by which proteins fail to attain or maintain their native conformation, often resulting in partially folded or misfolded states prone to self-assembly into abnormal aggregates [21]. While transient misfolding can be corrected by cellular quality control, persistent misfolding can lead to toxic gain-of-function aggregates or loss-of-function due to sequestration of essential proteins [22]. This section provides an overview of molecular mechanisms governing protein folding, quality control, and how their failure underlies protein aggregation in disease, with emphasis on neurodegenerative disorders.

### 3.1. Protein Folding and Molecular Chaperones

In cells, protein folding begins co-translationally and continues post-translationally with the aid of molecular chaperones. Chaperones such as the heat shock proteins (Hsp)—e.g., Hsp70, Hsp90, Hsp60—bind nascent or stress-unfolded polypeptides to prevent improper interactions and facilitate correct folding [23]. For instance, Hsp70 family chaperones use ATP-dependent cycles to stabilize unfolded proteins and give them another chance to fold [24], while Hsp90 often assists in the later stages of folding for signaling proteins and receptors [25]. Co-chaperones regulate chaperone activity, targeting specific client proteins or modulating the ATPase cycle of major chaperones [26]. Under conditions of stress (heat shock, oxidative stress, etc.), cells upregulate chaperone expression as part of the heat shock response, expanding the folding capacity to counteract increased misfolding [27]. However, if misfolded proteins accumulate beyond the handling capacity of chaperones, a state of “proteostasis stress” ensues [28].

### 3.2. Proteostasis and Protein Quality Control System

Cells have evolved elaborate proteostasis networks to maintain protein homeostasis, encompassing synthesis, folding, trafficking, and degradation [22]. Two major degradation systems ensure that misfolded or surplus proteins are cleared: the ubiquitin-proteasome system (UPS) and the autophagy-lysosome pathway [29].

In the UPS, misfolded or damaged proteins are tagged with ubiquitin chains by E1-E2-E3 enzyme cascades and targeted to the 26S proteasome, a protease complex that degrades ubiquitinated proteins into peptides. This system is critical for degrading short-lived and misfolded proteins, particularly in the cytosol and nucleus [30]. Key to UPS function is substrate recognition: chaperones and co-chaperones (like C-terminal Hsp70-interacting protein (CHIP), an E3 ubiquitin ligase) can triage terminally misfolded proteins for proteasomal degradation [31]. In neurodegenerative diseases, UPS impairment is commonly observed, leading to accumulation of ubiquitin-positive protein aggregates (as seen in Lewy bodies in PD or neurofibrillary tangles in AD) [32]. Interestingly, proteasome inhibition in experimental settings can reproduce features of neurodegeneration and even pain: for example, blocking proteasomes in sensory neurons induces neuropathic pain behaviors, highlighting the UPS’s role in neuronal function [33].

Autophagy is a process where cellular components, including protein aggregates and defective organelles, are engulfed in double-membrane vesicles (autophagosomes) that fuse with lysosomes for degradation [34]. There are multiple forms—macro autophagy, micro autophagy, and chaperone-mediated autophagy—but collectively, autophagy is crucial for clearing larger protein aggregates and damaged organelles, complementing the UPS [35]. In neurons, autophagy is vital due to their post-mitotic nature and polarized long processes; deficits in autophagy contribute to aggregate accumulation in AD (accumulation of autophagic vacuoles with Aβ), PD (impaired clearance of α-synuclein), and ALS (aggregates of SOD1 or TDP-43) [35,36]. Notably, chronic pain has been associated with autophagy changes as well; for instance, in diabetic neuropathy models, heightened protein kinase C ε (PKCε) activity led to increased autophagosome formation alongside ER stress [37], linking autophagy to pain mechanisms.

Secretory and membrane proteins fold in the endoplasmic reticulum (ER) [38]. The ER has its own quality control: misfolded proteins are recognized and retrotranslocated to the cytosol for degradation via ER-associated degradation (ERAD) pathways [39]. Accumulation of misfolded proteins in the ER lumen triggers the unfolded protein response (UPR) (discussed below) [40], which transiently reduces protein synthesis, increases chaperone production (like BiP/GRP78), and enhances ERAD capacity [41]. If ER misfolding is not resolved, UPR signaling can initiate apoptosis [42]. The UPR is particularly relevant in both NDs and chronic pain contexts, as we will explore.

### 3.3. Unfolded Protein Response (UPR) and ER Stress

The unfolded protein response (UPR) is a conserved cellular stress response to ER stress—a condition characterized by an accumulation of misfolded or unfolded proteins in the ER [40]. The UPR is orchestrated by three ER membrane sensors: inositol-requiring enzyme 1 (IRE1), protein kinase R-like ER kinase (PERK), and activating transcription factor 6 (ATF6) [43]. Under normal conditions, these sensors are kept inactive by binding to the ER chaperone binding immunoglobulin protein/78-kDa glucose-regulated protein (BiP/GRP78) [44]. When misfolded proteins accumulate, BiP dissociates from these sensors to assist in protein folding, thereby freeing and activating them [41]. IRE1 dimerizes and auto phosphorylates, gaining endoribonuclease activity that splices X-box binding protein 1 (XBP1) mRNA to produce spliced XBP1 (XBP1s), a transcription factor that upregulates UPR target genes involved in ERAD and chaperone production [45]. Additionally, IRE1 signaling can activate c-Jun N-terminal kinase (JNK) and nuclear factor kappa-light-chain-enhancer of activated B cells (NF-κB), thereby linking ER stress to inflammatory responses [46]. PERK phosphorylates eukaryotic initiation factor 2 alpha (eIF2α), transiently attenuating global protein translation to reduce the ER client load [47]. However, this paradoxically increases translation of activating transcription factor 4 (ATF4), another transcription factor that induces genes involved in redox balance, amino acid metabolism, and stress adaptation [48]. If ER stress persists, ATF4 also promotes the expression of C/EBP homologous protein (CHOP), a pro-apoptotic factor that triggers programmed cell death [49]. ATF6 translocates to the Golgi apparatus upon ER stress, where it is cleaved by site-1 protease (S1P) and site-2 protease (S2P), releasing a cytosolic fragment that functions as a transcription factor [50]. This active ATF6 fragment upregulates genes encoding ER chaperones and ERAD components, enhancing the cell’s ability to restore proteostasis [51].

In neurodegenerative diseases, markers of chronic UPR activation are often present, indicating unresolved ER stress [52]. For example, phosphorylated PERK and eIF2α are observed in postmortem brains of AD patients [53]. In PD, α-synuclein aggregates may directly cause ER stress by disrupting ER-Golgi trafficking and proteasome function [54]. Importantly, UPR is also implicated in pain: peripheral nerve injury or inflammation can induce ER stress in dorsal horn neurons and dorsal root ganglion cells, contributing to neuropathic pain states [55]. This connection between UPR and pain underscores the broader relevance of proteostasis across conditions.

### 3.4. Key Disease-Associated Proteins and Prion-like Propagation

Several proteins are considered critical “drivers” of pathology in major NDs due to their propensity to misfold and form aggregates. Below, we highlight the primary misfolded proteins implicated in AD, PD and ALS.

Aβ is produced from amyloid precursor protein (APP) via β- and γ-secretase cleavage [56]. Misfolded Aβ monomers form oligomers—considered the most synaptotoxic species—which can further aggregate into insoluble fibrils and extracellular plaques [57]. Tau is a microtubule-associated protein that stabilizes neuronal cytoskeleton; in disease, it becomes hyperphosphorylated and misfolds into paired helical filaments and neurofibrillary tangles [58]. Aβ and tau pathologies synergize: Aβ aggregates can seed tau misfolding in a prion-like manner, exacerbating neurotoxicity in AD [59]. Prion-like seeding refers to the phenomenon where a misfolded protein induces conformational change in a normal counterpart, spreading in a templated manner [60]. Both Aβ and tau have shown such properties: intracerebral injections of brain extract rich in Aβ or tau aggregates can induce plaques and tangles in transgenic mice [61].

α-Synuclein is a presynaptic protein involved in synaptic vesicle regulation [62]. In PD and related disorders, α-synuclein misfolds from its native unfolded state into β-sheet rich oligomers and fibrils, aggregating as Lewy bodies inside neurons [63]. Mutations (e.g., synuclein alpha (SNCA) gene multiplications or point mutations) and environmental factors promote α-synuclein misfolding [63]. These misfolded species can propagate trans-synaptically: studies show that pathological α-synuclein can transfer from affected to healthy neurons, likely via exosomes or nanotubes, seeding new aggregates [64]. This propagation may underlie the stereotypical spread of pathology in PD, from brainstem to cortex [65]. Importantly, α-synuclein pathology is not confined to the brain—aggregates in the spinal cord dorsal horn have been observed in PD patients with chronic pain [66], hinting at a link between α-synuclein and nociceptive processing.

In most ALS cases (sporadic and familial), mislocalized and aggregated TDP-43 (an RNA/DNA-binding protein) is a pathological hallmark [67]. TDP-43 normally resides in the nucleus, but in ALS, it misfolds, forms cytoplasmic inclusions, and loses nuclear function, impairing RNA processing [67]. Superoxide dismutase 1 (SOD1) mutations cause some familial ALS; mutant SOD1 misfolds and oligomerizes, triggering motor neuron toxicity via oxidative stress and possibly prion-like spread to neighboring cells [68]. FUS is another DNA/RNA-binding protein aggregating in certain ALS and frontotemporal dementia cases [69]. Notably, like prions, misfolded SOD1 has been shown to induce misfolding of wild-type SOD1 in vivo [70]. The ability of these proteins to self-propagate misfolded states positions protein aggregation as a driving mechanism that can disseminate pathology within the nervous system.

### 3.5. Neuroinflammation and Oxidative Stress in Protein Misfolding

Misfolded protein aggregates do not act in isolation; they trigger and are influenced by neuroinflammation and oxidative stress [71]. Activated microglia and astrocytes commonly surround protein aggregates in NDs, releasing pro-inflammatory cytokines (TNF, IL-1β, IL-6) and reactive oxygen/nitrogen species that can exacerbate protein misfolding [72]. For example, Aβ oligomers engage microglial receptors (like Toll-like receptors (TLRs) and NLR family pyrin domain containing 3 (NLRP3) inflammasome), leading to cytokine release, which in turn can promote tau phosphorylation and neuronal stress [72,73]. Chronic oxidative stress, as seen with aging or mitochondrial dysfunction, increases the pool of oxidatively damaged, misfolded proteins and impairs chaperone and proteasome function [74]. In PD, dopaminergic neurons are particularly susceptible to oxidative damage from dopamine metabolism and mitochondrial deficits; this environment fosters α-synuclein misfolding and aggregation [75]. Conversely, protein aggregates can directly cause oxidative stress by disrupting mitochondrial function (e.g., Aβ can damage mitochondrial membranes, α-synuclein can impair complex I of the electron transport chain) [76,77,78]. This creates a feed-forward loop: oxidative stress begets more misfolding, and misfolded aggregates generate more oxidative stress and inflammation.

Glial cells play a dual role. While acute activation may aid clearance of debris, chronic microglial activation can impair phagocytosis and create a chronic inflammatory milieu that overwhelms neurons’ proteostasis capacity [79]. Astrocytes under inflammatory conditions can lose some support functions (like glutamate uptake) and contribute to excitotoxic and proteotoxic stress on neurons [80]. Some misfolded proteins (e.g., SOD1) are now understood to be secreted in exosomes or as naked fibrils that can activate glia in a manner akin to pathogen-associated particles [81], further cementing the link between protein aggregation and neuroimmune activation.

**Figure 1:**
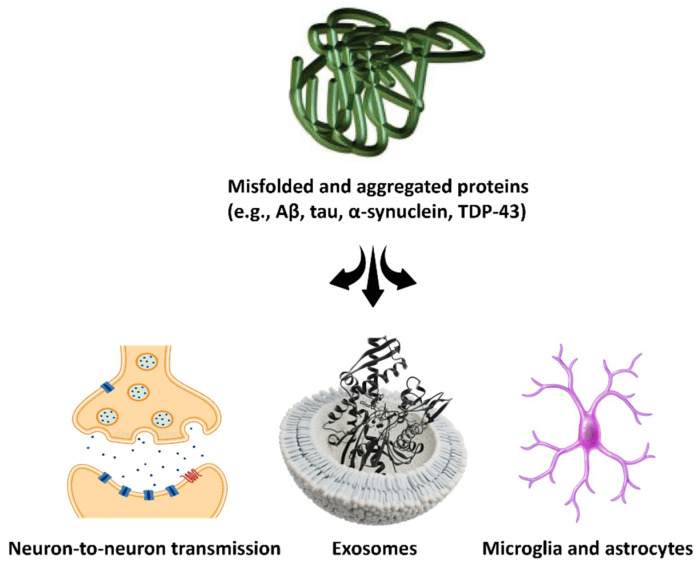
Prion-like propagation of misfolded proteins in pain and neurodegeneration. Misfolded and aggregated proteins propagate between neurons and glial cells through multiple mechanisms, resembling prion-like transmission. Pathological proteins can spread via (1) neuron-to-neuron transmission through axonal transport, (2) exosomal transfer between cells, and (3) microglial engulfment and re-release of misfolded proteins, among others. Neuroinflammation, particularly microglial activation and cytokine release, exacerbates protein propagation, contributing to neurodegeneration. Chronic pain may further accelerate this process by maintaining a prolonged inflammatory state, increasing oxidative stress, and disrupting proteostasis, ultimately amplifying misfolded protein accumulation and spread.

In summary, protein misfolding and aggregation in neurodegenerative diseases involve a network of molecular derangements: intrinsic factors of protein structure and folding, overwhelmed quality control systems (UPS, autophagy, UPR), prion-like spread of aggregates, and extrinsic factors like chronic inflammation and oxidative stress. These factors collectively drive progressive neuronal dysfunction and death. Given these common themes across NDs, a critical inquiry of this review is whether similar processes occur in chronic pain states and, if so, how they might contribute to a reciprocal relationship between chronic pain and neurodegeneration. Figure 1 represents the prion-like propagation of misfolded proteins in pain and neurodegeneration, highlighting the mechanisms of protein spread and the role of neuroinflammation.

## 4. Chronic Pain and Protein Misfolding: Mechanistic Insights

Emerging research indicates that chronic pain is not merely a symptom arising from static neural damage or peripheral input; it is accompanied by active biochemical and cellular changes in the nervous system, some of which mirror those seen in neurodegenerative conditions [82,83]. This section explores evidence that chronic pain—particularly neuropathic pain and other persistent pain states—is associated with protein misfolding and proteostasis disturbances. We discuss findings from animal models of chronic pain and human studies, detailing how prolonged nociceptive signaling, neuroinflammation, and cellular stress in pain conditions could induce protein aggregation or misfolding in neural tissue.

### 4.1. Evidence of Protein Aggregation in Chronic Pain Conditions

Historically, chronic pain has been studied in terms of synaptic plasticity and neural circuit sensitization [84]. However, several lines of evidence now point to protein aggregation and misfolding occurring in chronic pain states [85].

Parkinson’s Disease and Pain. PD offers a human illustration of pain-aggregation overlap. Over 30–50% of PD patients experience chronic pain (musculoskeletal, neuropathic, or central pain) during their illness [86]. Autopsy analyses reveal that α-synuclein-containing Lewy bodies are not confined to the brain; they also appear in the dorsal horn of the spinal cord in PD patients, particularly those with pain symptoms [87]. These Lewy bodies in pain-processing regions suggest that misfolded α-synuclein could directly or indirectly contribute to neuropathic pain in PD [88]. Additionally, experimental α-synuclein overexpression in rodent models has been linked to abnormal nociceptive processing, although research is ongoing to clarify this relationship [89].

Multiple Sclerosis and Pain. Multiple sclerosis (MS) is primarily an autoimmune demyelinating disease, but chronic neuropathic pain (e.g., dysesthetic pain) is common in MS patients [90]. Interestingly, in postmortem analyses of MS patients, increased levels of ER stress markers and misfolded protein responses were observed in dorsal root ganglia (DRG) neurons [91]. One study found that DRG of MS patients (who often report chronic pain) had heightened expression of BiP and other UPR markers, implying a proteostasis disturbance in peripheral sensory neurons [92]. Though MS is not classically a protein aggregation disease, this finding aligns with the idea that chronic pain conditions involve stress responses that overlap with those known to precipitate protein misfolding.

Peripheral Nerve Injury Models. In rodent models of neuropathic pain (such as spinal nerve ligation (SNL)), there is direct evidence of ER stress and UPR activation in the spinal cord. Zhang et al. (2015) reported that SNL in rats induced significant upregulation of BiP and spliced XBP1 in spinal dorsal horn neurons. These changes indicate an ongoing UPR due to misfolded protein accumulation in neurons after nerve injury [93]. Notably, treating SNL rats with a chemical chaperone, tauroursodeoxycholic acid (TUDCA), attenuated both the ER stress markers and the pain behavior [94]. TUDCA facilitates protein folding and ER stress resolution, suggesting that relieving proteostasis burden can alleviate neuropathic pain [95]. Similarly, intrathecal administration of thapsigargin (which induces ER Ca^2+^ depletion and misfolding) in healthy rats caused mechanical hyperalgesia, essentially phenocopying chronic pain via pure ER stress induction [96].

Diabetic Neuropathy. Chronic diabetic peripheral neuropathy is another condition marked by persistent pain (often burning or tingling sensations) [97]. High glucose and metabolic stress in diabetes can cause widespread protein misfolding stress, and indeed, diabetic neuropathy models show sustained UPR activation [98]. In diabetic mice, elevated PERK phosphorylation and CHOP expression have been detected in peripheral nerves [99]. Kan et al. (2024) demonstrated that in diabetic neuropathic pain, there is increased ER stress and autophagy, mediated by PKCε upregulation [37]. Use of a PKCε inhibitor reduced both ER stress markers and pain hypersensitivity [37]. Moreover, pharmacological chaperones like 4-phenylbutyric acid (4-PBA) can reverse diabetes-induced pain behaviors, reinforcing that a misfolding-driven mechanism is at play [100]. Taken together, these findings highlight proteostasis failure (ER stress, defective autophagy) as a contributor to diabetic pain.

Chronic Inflammatory Pain. Conditions like osteoarthritis (OA) and rheumatoid arthritis (RA) cause chronic pain and have systemic inflammatory components [101]. Chondrocytes and synoviocytes in arthritic joints show ER stress due to high secretory demand and inflammation, which can lead to misfolded collagen and other matrix proteins [102]. While the local joint pathology is well known, recent studies indicate that systemic inflammation in chronic pain can promote amyloid and tau changes in the brain [103]. For instance, chronic musculoskeletal pain in middle-aged individuals correlates with higher blood Aβ levels, especially in those with elevated C-reactive protein (CRP) [104]. Chronic pain plus systemic inflammation was associated with significantly higher plasma Aβ42 and Aβ40 in a cohort of older men, suggesting that chronic peripheral inflammation might dysregulate amyloid metabolism or production (possibly via platelet activation or blood–brain barrier permeability changes) [104]. Although not direct evidence of central nervous system (CNS) aggregation, it hints at a link between chronic pain/inflammation and amyloidogenic processing.

Fibromyalgia and Central Sensitization Syndromes. Fibromyalgia (FM) is characterized by widespread chronic pain with features of central sensitization and dysregulated pain processing [105]. Some emerging hypotheses suggest that chronic stress and metabolic strain in FM could impair mitochondrial function and increase oxidative stress in the CNS [106]. There is limited direct evidence of protein aggregation in FM, but one study found elevated levels of cerebrospinal fluid tau in FM patients, analogous to what is seen in early AD, though interpretation is complex (tau can increase with neuroinflammatory or neurodegenerative processes broadly) [107]. More robust is evidence of glial activation in FM and chronic low back pain via TSPO PET imaging, which, while not a direct measure of misfolding, indicates an environment in which misfolding could be more likely due to chronic cytokine exposure [108].

In summary, chronic pain conditions, especially those with neuropathic components, show clear evidence of proteostasis disturbances: from ER stress and UPR activation to benefits of chemical chaperones and presence of misfolded protein aggregates in pain-related anatomical regions. These findings support the notion that chronic pain is not just a functional state but involves molecular pathology that overlaps with protein misfolding diseases.

### 4.2. Proteostasis Dysfunction and Neuronal Stress in Chronic Pain

Persistent Neuronal Firing and Metabolic Stress. Chronic pain may lead to protein misfolding and aggregation due to the cellular stress induced by prolonged high-frequency neuronal activity and persistent inflammation within pain pathways [109]. Chronic pain often involves hyperexcitability of pain pathways (e.g., dorsal horn neurons or cortical pain matrices exhibit increased spontaneous activity and responsiveness) [110]. Sustained neuronal firing can stress neuronal metabolism, particularly in mitochondria. Neurons are high-energy cells, and continuous activity can lead to mitochondrial dysfunction and Ca^2+^ dysregulation [111]. Mitochondrial dysfunction is a known trigger for increased production of reactive oxygen species (ROS) and impaired ATP production, both of which hamper protein folding (less energy for chaperones, more oxidative damage to proteins) [112]. There is evidence that in models of chronic inflammatory pain, such as persistent inflammation induced by Complete Freund’s Adjuvant, sensory neurons undergo mitochondrial changes that outlast the acute phase. Such changes include altered mitochondrial membrane potential and increased ROS generation, which can activate the UPR and other stress responses [113].

Calcium Dysregulation. Neuronal excitability and ER stress intersect at calcium. The ER is a major Ca^2+^ store, and many ER chaperones are Ca^2+^-dependent [114]. Overactivation of neurons (as in chronic pain central sensitization) can perturb Ca^2+^ homeostasis both in the cytosol and ER. If ER Ca^2+^ stores are depleted by continuous signaling, protein folding in the ER suffers, triggering UPR activation [115]. Indeed, pharmacologically inducing ER Ca^2+^ release (with agents like thapsigargin) causes pain behaviors, as mentioned [96]. Chronic pain conditions might similarly involve calcium dysregulation that promotes protein misfolding [116].

Chronic UPR Activation. As noted, studies show chronic pain models have sustained UPR activation [93,98]. Normally, the UPR aims to restore equilibrium, but if the cause (e.g., nerve injury) persists, the UPR remains chronically active, which can lead to UPR maladaptation [117]. This state can reduce overall protein synthesis (via PERK-eIF2α) and alter synaptic protein expression, potentially contributing to synaptic reorganization seen in chronic pain (e.g., loss of inhibitory interneurons or changes in receptor trafficking) [118,119]. Chronic UPR may also tip into pro-apoptotic signaling via CHOP, possibly contributing to neurodegeneration in long-standing pain [49].

Glial and Neuroimmune Factors. In chronic pain, microglia and astrocytes in the spinal cord and brain are persistently activated, releasing cytokines (TNF, IL-1β, IL-6), chemokines, and growth factors that modulate neuronal excitability [120]. These factors, while key to central sensitization, also impose stress on neurons. For example, TNF has been shown to induce ER stress through ROS-mediated pathways [121]. In a broader sense, chronic neuroinflammation can lead to a state called “inflammation-induced proteotoxicity” where the protein quality control machinery is overwhelmed by inflammatory damage [122]. Therefore, the neuroimmune activation in chronic pain is a plausible contributor to protein misfolding.

Nociceptive Plasticity and Protein Synthesis. Long-term potentiation (LTP)-like phenomena occur in pain pathways (e.g., at C-fiber synapses in the dorsal horn) [123]. Such synaptic plasticity often requires new protein synthesis, which if dysregulated could produce misfolded proteins [124]. Some studies suggest that aberrant local protein synthesis in neurons contributes to pain memory (e.g., protein kinase Mζ in maintaining chronic pain) [125]. If this local translation is excessive or in the presence of stress (inflammation, ROS), it might produce misfolded proteins that aggregate locally at synapses. Additionally, one aspect of central sensitization is altered expression of ion channels and receptors (e.g., increased Nav1.8 sodium channel expression in DRG, increased N-methyl-D-aspartate (NMDA) receptors in spinal cord) [126,127]. Upregulation of these proteins can tax the ER folding machinery, again linking back to UPR [128].

### 4.3. Chronic Pain as a Risk Factor for Protein Misfolding

Beyond mechanistic overlaps, chronic pain itself may be a risk factor for developing neurodegenerative pathology through protein misfolding, as evidenced by several key points discussed earlier [129]. Chronic pain is associated with approximately 1.5- to 2-fold increased risk of developing AD-related dementias [130]. It is unlikely that pain simply co-occurs by chance; instead, persistent pain may contribute to or exacerbate pathology, although direct causality in humans remains to be fully established. The mouse model by Guerreiro et al. (2022) clearly showed chronic pain was associated with tau-dependent hippocampal degeneration in animal models [17]. This suggests that, in this animal model, chronic pain may precede tauopathy development. A Mendelian Randomization (MR) study suggested a potential directional association between chronic pain and increased AD risk using genetic instruments [131]. While MR has assumptions and limitations, it bolsters the epidemiologic association by hinting at a directional influence of pain on dementia. This suggests that something about chronic pain biology (potentially chronic stress or inflammation) is driving AD pathology rather than vice versa.

As repeatedly emphasized, chronic pain leads to chronic neuroinflammation (both central and peripheral) [132]. Neuroinflammation is a well-known precipitant of protein misfolding and aggregation [133]. For example, injections of lipopolysaccharide (LPS) to induce inflammation in animals can precipitate amyloid and tau pathology [134]. Chronic pain patients often have elevated systemic markers like CRP, and higher CRP in pain patients was linked to higher plasma Aβ (perhaps from platelets) [135]. This aligns with models where peripheral inflammation feeds neurodegeneration via circulating factors and blood–brain barrier penetration [136]. Hence, chronic pain might accelerate neurodegeneration by keeping the brain’s immune environment in a prolonged activated state that favors protein aggregation.

Chronic pain can indirectly increase risk factors for misfolding diseases. For example, poor sleep (common in chronic pain) can reduce glymphatic clearance of Aβ in brain interstitial fluid, leading to accumulation [137]. Depression and chronic stress, frequent companions of pain, raise cortisol and glutamate levels that can damage neurons and proteins [138]. Opioid use, while relieving pain, has been linked to exacerbating neuroinflammation and even UPR activation (as seen in opioid-induced hyperalgesia where morphine triggered the UPR in glia) [139]. These factors interplay to tip the balance toward proteostasis breakdown.

In summary, mechanistic insights reveal that proteostasis dysfunction is both a consequence and a contributor to chronic pain. Chronic pain states impose ER stress, mitochondrial stress, and neuroinflammation that collectively can drive protein misfolding and aggregation in neural tissues. This not only suggests a molecular kinship between chronic pain and neurodegenerative diseases but also implicates chronic pain as a potential contributor to the initiation or amplification of similar pathogenic cascades that later manifest as neurodegeneration. Recognizing chronic pain as more than a symptom—as a state that can precipitate molecular neuropathology—is a paradigm shift with significant implications for how we monitor and treat individuals with persistent pain.

## 5. Neurodegeneration and Chronic Pain: Common Pathways

Building on the evidence that chronic pain involves protein misfolding and that neurodegenerative diseases share inflammatory and proteostatic features, we now delve into the common molecular and cellular pathways that may underlie both chronic pain and neurodegeneration. Understanding these shared pathways can clarify why these conditions co-occur and how they might exacerbate each other.

### 5.1. Shared Neuroinflammatory and Neuroimmune Mechanisms

Glial Activation. Both chronic pain and neurodegenerative diseases are marked by persistent activation of glial cells—microglia and astrocytes—in the central nervous system [140,141]. In chronic pain (especially neuropathic pain), microglia in the spinal cord dorsal horn transition to a reactive state, releasing pro-inflammatory mediators that enhance pain signaling [142]. Astrocytes in chronic pain can become hypertrophic and secrete substances that sustain neuronal hyperexcitability [143]. Similarly, in NDs, microglia and astrocytes respond to accumulating protein aggregates and dying neurons, often entering a chronic inflammatory state that fails to resolve the pathology and instead contributes to progression [141]. For instance, microglial activation is observed early in AD (even before overt plaques) and is believed to drive a feed-forward cycle of Aβ production and synaptic damage [144]. In ALS, microglia initially attempt to clear neuron debris but later become neurotoxic, releasing superoxide and proteases that harm motor neurons [145]. Thus, chronic microglial activation is a hallmark of both pain and neurodegeneration.

Cytokines and Chemokines. There is substantial overlap in the inflammatory cytokines elevated in chronic pain and NDs. TNF, IL-1β, and IL-6 are perhaps the most well-documented. In chronic pain, elevated levels of TNF and IL-1β have been measured in CSF or affected nerves (e.g., in sciatica, disk herniation, or peripheral neuropathy) [146]. These cytokines enhance pain by various means (sensitizing nociceptors, reducing inhibitory neurotransmission, etc.) [147]. In neurodegeneration, TNF and IL-1β are found at increased levels in brains of AD and PD patients and correlate with disease severity [148,149]. IL-6, a cytokine with both pro- and anti-inflammatory roles, is elevated in systemic circulation of chronic pain patients (e.g., chronic low back pain) and in AD brain and CSF [150,151]. Chronic pain also involves chemokines like C-C motif ligand 2 (CCL2), which recruits microglia/macrophages [152]; the same chemokine is implicated in AD for recruiting peripheral immune cells to the brain [153]. Therefore, the neuroimmune crosstalk—involving cytokine and chemokine signaling—is a shared pathway that can lead to synaptic dysregulation and neuronal damage in both scenarios.

Toll-like Receptors (TLRs) and Danger Signals. Misfolded proteins and nerve injury can both act as “danger signals”. TLR4, for example, is a receptor on microglia that mediates pain hypersensitivity when activated by damage-associated molecular patterns (DAMPs) [154]. Hemoglobin from nerve injury or high-mobility group box 1 (HMGB1) released by stressed neurons can activate TLR4, contributing to pain and neuroinflammation [155]. Interestingly, TLR4 is also implicated in AD—Aβ can bind to TLR4 on microglia, initiating inflammatory cascades [156]. Similarly, misfolded α-synuclein can activate TLR2 and TLR4 [157], and mutant SOD1 in ALS can engage TLR signaling [158]. These innate immune pathways are thus common to chronic pain and NDs, as both involve persistent “danger” signals that keep the immune system activated chronically.

**Figure 2:**
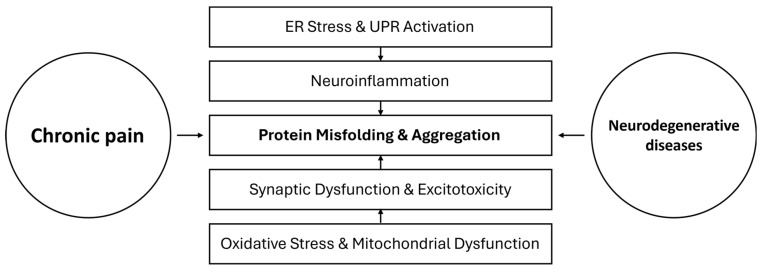
Mechanistic pathways linking chronic pain to neurodegenerative diseases. Chronic pain and neurodegenerative diseases share interconnected pathophysiological mechanisms that drive progressive neuronal dysfunction. These mechanisms are closely linked and may converge at protein misfolding and aggregation. Chronic pain can aggravate cellular stress responses, leading to proteostasis disruption and neurodegeneration, while neurodegenerative processes may increase pain sensitivity, thereby establishing a bidirectional loop.

NLRP3 Inflammasome. Another immune mechanism in common is the NLRP3 inflammasome, a protein complex in microglia and macrophages that activates caspase-1 to produce IL-1β and IL-18 [159]. In chronic pain, particularly neuropathic pain, the NLRP3 inflammasome in spinal microglia has been shown to contribute to pain hypersensitivity [160]. Likewise, in AD, NLRP3 is activated by Aβ and is thought to exacerbate amyloid pathology by sustaining microglial inflammation [161]. Mitochondrial dysfunction (present in both pain and NDs) can trigger NLRP3 via release of mitochondrial DNA or ROS [162]. Thus, inflammasome activation is another convergent point. Figure 2 represents the mechanistic pathways linking chronic pain to neurodegenerative diseases, illustrating the interconnected pathophysiological mechanisms that drive neuronal dysfunction and the bidirectional cycle between pain and neurodegeneration.

### 5.2. Dysregulation of Protein Homeostasis in Pain and NDs

We have previously detailed how chronic pain can cause proteostasis. Neurodegenerative diseases by definition involve proteostasis failure leading to aggregates.

Autophagy Impairment. Reduced autophagic flux is seen in models of neuropathic pain (e.g., chronic constriction injury leads to accumulation of autophagosomes in DRG neurons, indicating possible block in autophagosome-lysosome fusion) [163]. In neurodegeneration, autophagy impairment is near-universal (AD neurons show accumulated autophagic vacuoles, PD has mutations in autophagy genes like leucin rich repeat kinase (*LRRK2*, ALS in *C9orf72* can impair autophagy) [164,165,166]. Rapamycin, an autophagy enhancer, has been tested both to reduce protein aggregates in neurodegeneration and to alleviate pain in some chronic pain models, highlighting a functional intersection: boosting autophagy might benefit both [167,168,169,170].

Proteasome and Ubiquitin System. After nerve injury, there is evidence of changes in the UPS in the dorsal horn—some studies show downregulation of proteasome subunits in chronic pain models, which parallels the proteasome impairment observed in ND models and patient tissues [171]. Conversely, proteasome inhibitors (like bortezomib used in cancer) cause peripheral neuropathy as a side effect, demonstrating that UPS inhibition can produce pain [172]. This is akin to the ND context where proteasome inhibition leads to aggregate accumulation and cell death [173]. Thus, the UPS’s role in pain and ND might be two sides of the same coin—proteasome dysfunction can cause pain (via accumulating signaling molecules or failing to remove damaged proteins in nociceptors) and cause ND (via aggregate build-up).

Mitochondrial Quality Control. Both chronic pain and NDs involve mitochondrial stress. In PD, for example, Parkin and PTEN-induced kinase 1 (PINK1) mediate mitophagy (clearance of damaged mitochondria), and their mutation leads to PD [174]. In chronic pain, one study showed that a TSPO ligand (which modulates mitochondrial function) alleviated neuropathic pain, implying mitochondrial involvement [175]. If chronic pain is causing slight mitochondrial damage continuously (due to glutamate excitotoxicity or inflammation), and if that overwhelms mitophagy, neurons might accumulate dysfunctional mitochondria, a situation also present in aging and NDs. This could be another shared path: impaired mitochondrial proteostasis (i.e., inability to maintain healthy mitochondria and mitochondrial proteins) linking pain and degeneration.

### 5.3. Prion-like Propagation of Protein Aggregates in Pain and NDs

One intriguing area is whether prion-like mechanisms are active in chronic pain. In NDs, the prion-like spread of Aβ, tau, α-synuclein, and others is an area of intense research. Could chronic pain involve something akin to prion-like processes?

Some hypotheses propose that chronic pain could involve spread of dysfunctional proteins or signals along pain pathways [55]. For example, after a peripheral nerve injury, chemokines and perhaps misfolded channels might travel up the neuraxis. In a rodent model of PD, misfolded α-synuclein injected into gut or muscle can propagate to the brain and also to the spinal cord dorsal horn [176]. If α-synuclein or tau pathology is present in a person, intense nociceptive activity could hypothetically facilitate its spread into pain circuits due to activity-dependent release or exosomal transfer. Conversely, chronic pain’s inflammation might cause local protein modifications that then propagate. While direct evidence of a “prion-like” chronic pain protein is lacking, it is notable that tau pathology has been reported in the spinal cord in some conditions and that tau can propagate from cord to brain in models of traumatic injury [177]. Given that chronic pain can be initiated by traumatic nerve injuries, one could speculate that misfolded tau or other proteins at the injury site might travel to the brain, possibly contributing to central changes.

Exosomes and Extracellular Vesicles. Exosomes are emerging as key mediators of intercellular communication in both pain and NDs [178,179]. Neurons and glia release exosomes containing proteins (including misfolded ones) and RNA [180]. In AD and PD, exosomes can carry Aβ, tau, and α-synuclein and potentially seed pathology in recipient cells [181,182,183]. In chronic pain, recent studies have shown that exosomes derived from injured nerves or inflamed tissues can induce pain by transferring cargo to neurons and glia. For example, exosomes from arthritic joints carry inflammatory mediators that sensitize neurons [184]. Additionally, circulating exosomes in patients with complex regional pain syndrome have distinct protein cargo profiles [185]. This suggests that exosomes could conceivably carry misfolded proteins or stress signals from peripheral injury sites to the central nervous system, akin to how cancer exosomes prepare metastatic niches [186]. The role of exosomes in transmission is a promising commonality: targeting exosome release or uptake is being explored to slow neurodegeneration and could also be a strategy to reduce pain propagation [187,188].

### 5.4. Impact of Chronic Pain on Neurodegenerative Progression

Clinical Observations. Patients with established neurodegenerative diseases often report that pain worsens their neurological symptoms. For example, AD patients with untreated pain show more pronounced behavioral disturbances and cognitive decline, possibly due to pain-related stress and inflammation [14]. PD patients with chronic pain tend to have lower quality of life and possibly faster mobility decline [189]. These observations hint that pain management could be an important aspect of caring for ND patients, not just for comfort but potentially for slowing disease.

Imaging Studies. Neuroimaging is starting to capture how chronic pain might accelerate neurodegeneration. A study using magnetic resonance imaging (MRI) over several years found that individuals with chronic low back pain had a greater rate of gray matter atrophy in the dorsolateral prefrontal cortex and hippocampus than those without pain [190]. The hippocampus is notably vulnerable in AD, and indeed, in one report, the volume loss in hippocampi of people with chronic pain was equivalent to several additional years of brain aging compared to controls [191]. This suggests chronic pain contributes to brain atrophy in regions overlapping ND processes (hippocampus for memory, prefrontal for executive function).

Biomarker Studies. The 2023 study [103] found that among individuals with biomarker evidence of non-AD pathology (suspected non-Alzheimer’s pathophysiology), those with chronic pain had higher CSF total tau and soluble triggering receptor expressed on myeloid cells 2 (sTREM2) (a microglial activation marker) than those without pain. They also had higher CSF TNF [103]. This indicates that even in preclinical or atypical ND cases, pain associates with more intense neurodegenerative changes in CSF. In contrast, those with typical AD pathology plus pain did not show as clear a difference, possibly because the AD pathology was dominant [103]. Nonetheless, it underscores that pain can modulate classical ND biomarkers.

Neuroplasticity vs. Neurodegeneration. One might wonder if the neural changes in chronic pain (like synaptic reorganization or loss of certain inhibitory interneurons in the dorsal horn) constitute a form of neurodegeneration. While not equivalent to the widespread cell death in AD or ALS, chronic pain does involve loss or dysfunction of neuron subpopulations (e.g., loss of GABAergic interneurons in spinal cord has been noted in neuropathic pain, akin to a localized degeneration) [192]. The distinction blurs when considering that these changes might predispose to or interact with broader degenerative processes. For instance, if chronic pain causes loss of inhibitory tone in the hippocampus (through stress hormones or inflammation), it might make that network more susceptible to epileptiform activity or excitotoxic injury, which could worsen tau pathology.

In summary, chronic pain and neurodegenerative diseases share a host of pathological pathways: chronic glial activation, release of pro-inflammatory cytokines, persistent UPR and oxidative stress, impaired autophagy/UPS, and potentially prion-like spread of misfolded proteins via cell-to-cell transfer or exosomes. Chronic pain can be conceptualized as a state of chronic neural stress that may lower the threshold for neurodegeneration or accelerate ongoing degenerative processes. Recognizing these commonalities is crucial for developing holistic treatments that address both pain and neurodegenerative pathology, which we discuss next.

## 6. Potential Therapeutic Implications

Targeting protein aggregation in chronic pain management is an emerging therapeutic approach that warrants further investigation. Specific targets within this process, including molecular chaperones, components of autophagy and proteasomal degradation, and glial inhibitors, offer potential avenues for modulating pain. By leveraging diverse mechanisms to regulate protein misfolding pathways, these strategies may provide novel pain relief options, highlighting the need for continued research in this area.

Table 1 summarizes the most promising and therapeutically significant findings on protein misfolding modulation in neurodegenerative disorders, highlighting their potential implications for chronic pain management.

### 6.1. Chaperone Inhibitors

Heat shock protein 70 (Hsp70) and 90 (Hsp90) inhibitors have not been widely investigated for clinical pain management in humans. However, preclinical evidence suggests their potential role in modulating pain, particularly in neuropathic and inflammation-associated pain.

Hsp70 inhibitors, in particular, have shown promise in animal models, especially in the context of cancer pain. Krukowski et al. demonstrated that pifithrin-μ (PFM-μ), a dual p53 and chaperone inhibitor [200], prevents both paclitaxel- and cisplatin-induced peripheral neuropathy in chemotherapy-treated mice, thereby mitigating mechanical allodynia [195]. Further supporting this, Maj et al. highlighted the role of PFM-μ in preventing mitochondrial p53 aggregation in neuronal cells, offering additional evidence for its potential therapeutic application in cisplatin-induced neuropathy [201]. PFM-μ has demonstrated not only pain prevention in cancer mouse models but also neuroprotective effects in spinal cord injury models. When administered as a single dose in the early stages of injury, it exhibited anti-inflammatory properties by modulating microglial function [202], suggesting its potential for broader neurological applications. Hsp90 acts as a crucial cofactor in TLR4-mediated pain modulation by enhancing neuropathic pain and suppressing morphine analgesia, as its presence is required for TLR4 signaling to induce spinally mediated pain enhancement [203]. A study by Stine et al. [196] showed that Hsp90 inhibitors prevent opioids from exerting their anti-nociceptive effects in mouse models of chemotherapy-induced neuropathy and cancer-related bone pain. Furthermore, a 2021 systematic review and meta-analysis suggest that Hsp90 inhibitor treatment may increase the risk of pain in cancer patients, though the evidence linking Hsp90 inhibition to pain remains moderate [204]. Therefore, the role of Hsp90 protein in pain modulation remains elusive and its role in potential pain management is yet to be fully understood.

As of now, there are currently no FDA-approved Hsp70 or Hsp90 inhibitors for pain management. Although preclinical trials show promise, clinical trials for neuropathic, inflammatory, and cancer pain are still lacking. The current clinical data do not support the use of Hsp90 inhibitors as analgesics; instead, they may have adverse effects related to pain. Further research is needed to explore the potential of Hsp70 and Hsp90 inhibitors in pain management, including well-designed clinical trials focusing on their analgesic properties.

### 6.2. Modulation of Autophagy and Proteasomal Degradation

Chaperone-mediated autophagy, macro autophagy, and proteasomal degradation via the ubiquitin-proteasome system (UPS) are critical for the clearance of misfolded proteins [35]. Dysfunction in these pathways can lead to the accumulation of misfolded proteins, contributing to cellular stress and neurotoxicity. Autophagy and proteasomal degradation play essential roles in maintaining neuronal homeostasis by degrading misfolded proteins, eliminating damaged organelles, and regulating apoptosis and inflammation [205,206]. Their impairment has been well-documented in neurodegenerative disorders [35], suggesting they may also have a significant role in pain modulation through neuronal pathways.

Studies indicate that autophagy plays a crucial role in nerve damage and neuropathic pain development [207]. Animal models have demonstrated altered autophagic activity in the spinal cord, injured nerves, and brain following nerve injury [208,209,210]. Notably, upregulating autophagy has been shown to directly alleviate pain in some of these studies [209,211]. Additionally, autophagy may contribute to inflammatory pain modulation by inhibiting proinflammatory cytokine activity [212]. Agents that enhance autophagy and suppress inflammation, such as rapamycin, have demonstrated neuroprotective effects in preclinical models of neurodegenerative diseases, including AD, PD, and HD [213,214,215]. Rapamycin has also been shown to reduce pain symptoms in a chronic experimental autoimmune encephalomyelitis model, though its precise mechanism in treating neuropathic pain remains unclear [198]. Other autophagy stimulators, such as metformin, an adenosine monophosphate activated protein kinase (AMPK) activator, have shown promise in treating various chronic pain conditions, including diabetic neuropathy and fibromyalgia-type pain [216]. Additionally, trehalose, a fungal-derived sugar that induces autophagy via transcription factor EB (TFEB) activation, has been found to alleviate neuropathic pain in mice, though at the cost of reduced alertness [199].

The role of the UPS has been well established, as it is shown to have an impact on the development of chronic pain [171]. Studies have shown that proteasome inhibitors, such as epoxomicin, inhibitor of chymotrypsin-like catalytic site of the proteasome as well as the trypsin-like and the caspase-like sites, not only prevented development but also reversed nerve injury-induced pain behavior [217]. Similar results have been observed in models of inflammatory pain, as MG132, a synthetic reversible proteasome inhibitor, has shown promising results in suppression of pain and joint destruction in experimental osteoarthritis models [218]. Additionally, an interesting study by Gao et al. [194] has shown that a very common medication, aspirin, well known for its analgesic properties, decreases the pain while inhibiting proteasome degradation and promoting protein aggregate clearance through K63 ubiquitination.

Autophagy and proteasomal degradation play crucial roles in maintaining cellular homeostasis, and their dysfunction has been increasingly linked to the persistence of chronic pain. Modulating these pathways presents a promising therapeutic avenue, with preclinical studies demonstrating encouraging results in alleviating pain through enhanced protein clearance and reduced neuroinflammation. However, while these findings are compelling, further well-designed clinical studies are essential to determine the safety, efficacy, and long-term implications of targeting these mechanisms in chronic pain management.

### 6.3. Glial Inhibitors

Glia cells have a prominent role in the maintenance of central nervous system homeostasis, and microglial production of immune factors is believed to play an important role in nociceptive transmission [219]. Following peripheral nociceptive activation via nerve injury, microglia become activated and release pro-inflammatory cytokines such as TNF, IL-1β, and IL-6, thereby initiating the pain process [220]. Certain glial inhibitors, such as minocycline, have been shown to reduce protein aggregation in neurodegenerative disorders by inhibiting microglial activation and modulating inflammatory responses, which are key contributors to neuronal damage. Preclinical studies suggest that minocycline can decrease tau and amyloid-beta aggregation, as well as mitigate neuroinflammation, making it a promising therapeutic candidate for diseases characterized by protein misfolding [197]. Another glial inhibitor shown to reduce protein accumulation in neurodegenerative diseases is ibudilast, a potent phosphodiesterase inhibitor. A 2020 study demonstrated that ibudilast exerts neuroprotective effects not only by inhibiting glial activation but also by promoting autophagy and enhancing the clearance of disease-associated TDP-43 and SOD1 aggregates [221]. Sephin1, a novel glial inhibitor that prevents the dephosphorylation of eukaryotic initiation factor 2 alpha (eIF2α), has demonstrated neuroprotective effects in animal models of amyotrophic lateral sclerosis, Charcot-Marie-Tooth disease, multiple sclerosis, and other protein misfolding disorders [222]. While glial inhibitors have shown promise in neuroprotection and mitigating protein aggregation in neurodegenerative disorders, their potential role in pain conditions associated with protein misfolding remains to be elucidated through future preclinical and clinical investigations.

### 6.4. Antioxidant Therapy

The role of oxidative stress in neurodegenerative disorders is well established, with misfolded protein aggregation in diseases such as AD, PD, and HD being linked to the formation of ROS [223]. Given that both protein misfolding and oxidative stress contribute to chronic pain conditions, antioxidant therapeutic agents may hold promise for pain management. One such agent, N-acetylcysteine (NAC), a dietary supplement known for replenishing glutathione, has been shown to prevent the toxic effects of aggregated proteins by modulating heat shock protein activity, even independently of glutathione [193,224]. Additionally, NAC has demonstrated protective effects on glial cells against proteasome inhibitor toxicity without increasing glutathione levels [225]. Another antioxidant, resveratrol, has shown potential in suppressing protein aggregation in animal models, where dietary phosphorylated resveratrol improved locomotor abilities and physiological protein aggregation [226]. Furthermore, resveratrol has been reported to inhibit amyloid β42 and α-synuclein aggregation while reducing oxidative stress in vitro, highlighting its potential in treating neurodegenerative diseases linked to protein accumulation [227]. Curcumin, a bioactive compound found in turmeric, has also been shown to modulate protein aggregation and mitigate associated toxicity, potentially delaying ND onset by reducing endoplasmic reticulum stress [228,229].

Mitochondrial antioxidants, such as compounds like MitoQ (mitochondria-targeted coenzyme Q10) or SS peptides (small peptide antioxidants targeting the mitochondrial membrane), could in theory protect neurons in both chronic pain and ND by limiting mitochondrial ROS. For example, MitoQ improved neuropathic pain in a diabetic rat model by preserving mitochondrial function in nerves [230]. In AD models, MitoQ reduced oxidative damage and Aβ load [231].

While not exactly drugs, lifestyle modifications could also be useful in chronic pain management. Encouraging exercise, which upregulates endogenous antioxidant defenses [232] and chaperones [233], might help both chronic pain and has neuroprotective effects in aging and ND. Certain reviews suggest that diets containing polyphenols (like curcumin, epigallocatechin gallate (EGCG) from green tea) could have aggregation-inhibiting and antioxidant properties [234], potentially benefiting both groups of patients.

Despite substantial preclinical evidence supporting the role of antioxidants in mitigating oxidative stress and protein misfolding, clinical studies evaluating their effects on nociception in humans remain lacking. Thus, their potential role in pain management through the inhibition of protein aggregation remains uncertain.

### 6.5. Future Directions

The growing recognition of protein misfolding and aggregation in chronic pain conditions presents new opportunities for therapeutic intervention. By targeting these pathological aggregates through diverse pharmacological strategies, novel treatment paradigms may emerge that not only alleviate chronic pain but also address its underlying molecular mechanisms. Additionally, emerging evidence indicates that repurposing existing drugs could provide effective therapeutic options for managing chronic pain associated with protein misfolding disorders. Memantine, an NMDA receptor antagonist primarily used for Alzheimer’s disease, has demonstrated potential in alleviating neuropathic pain by reducing central sensitization. Its mechanism involves attenuating excitotoxicity, which may indirectly decrease neuronal stress related to protein misfolding [235]. Duloxetine, a serotonin and norepinephrine reuptake inhibitor approved for conditions like fibromyalgia and neuropathic pain, not only enhances mood but also exhibits neuroprotective properties. Studies indicate that duloxetine increases brain-derived neurotrophic factor (BDNF) levels, potentially supporting protein homeostasis and cognitive function [236]. Additionally, cannabinoids are under investigation for their analgesic and neuroprotective effects. Activation of CB2 receptors by cannabinoids has been shown to reduce microglial activation, thereby decreasing neuroinflammation in animal models of neurodegenerative diseases [237]. While clinical use of cannabinoids in dementia is limited due to side effects, their application in pain management is more prevalent.

Recognizing the link between chronic pain and neurodegeneration offers the potential to identify biomarkers that signal neurodegenerative risk, enabling earlier intervention. Measuring inflammatory markers such as serum CRP or IL-6 in chronic pain patients could help stratify those at higher risk of cognitive decline, allowing targeted anti-inflammatory or antioxidant therapies. Proteomic and exosomal markers may soon provide further insights, as CSF or blood tests detecting elevated misfolded proteins, such as increased CSF tau or plasma NfL, could indicate proteostasis stress, with high-throughput proteomics identifying specific signatures like heat shock or ubiquitinated proteins as red flags. Advanced imaging, including TSPO PET, could pinpoint glial activation in chronic pain patients, identifying those with central inflammation who may benefit from early microglial modulators, while functional MRI might reveal signs of accelerated brain aging, suggesting opportunities for intervention.

Exploring therapeutic implications at the intersection of chronic pain and neurodegenerative disease reveals a rich landscape. Approaches that stabilize protein folding, enhance clearance of aggregates, and quell chronic inflammation hold promise to treat pain more effectively and possibly delay neurodegenerative changes. It encourages more integrated management: treating chronic pain not only for immediate relief but as a preventive strategy for long-term neurological health. Conversely, aggressive management of mid-life pain could become part of dementia prevention protocols.

## 7. Conclusions

Chronic pain and neurodegenerative diseases, two of the most challenging domains in medicine, have traditionally been treated as separate entities. This review underscores a paradigm shift: chronic pain is not only a consequence of certain neurological conditions but may also actively contribute to neurodegenerative processes through shared mechanisms of protein misfolding and aggregation. We synthesized evidence that chronic pain can induce cellular stresses—including ER stress, mitochondrial dysfunction, and chronic neuroinflammation—that mirror those driving protein aggregation in classic neurodegenerative diseases like AD, PD, and ALS. Misfolded proteins and proteostasis deficits are emerging as a potential mechanistic bridge between sustained pain and progressive neurodegeneration.

Chronic pain states show activation of the unfolded protein response and accumulation of misfolded protein markers in both peripheral and central nervous system structures. Animal models link neuropathic pain with protein aggregation, where alleviating misfolding stress (via chaperones or UPR modulators) can reduce pain behaviors. Clinically, patients with chronic pain have higher risks of cognitive decline and exhibit biomarker changes (e.g., elevated tau, amyloid, and microglial markers) suggestive of an accelerated neurodegenerative process. Conversely, neurodegenerative disease models and patients often have heightened pain sensitivity or chronic pain, potentially due to overlapping neuroimmune and proteostatic disturbances. Glial cells stand out as central players in this interplay: microglial and astrocytic activation links pain and protein aggregation, propagating inflammatory and oxidative stress that damages neurons in a self-perpetuating loop.

These insights encourage a more holistic approach to patients. For clinicians, aggressive management of chronic pain could be important not just for quality of life, but also as a strategy to mitigate long-term neurodegenerative risk. Pain should be assessed in patients with neurodegenerative diseases, as untreated pain may hasten neurological decline. Multidisciplinary care—involving pain specialists, neurologists, and geriatricians—may be needed for patients at this intersection.

For researchers, this convergence opens exciting avenues. Could drugs that reduce protein aggregation in AD (like anti-amyloid or anti-tau therapies) also alleviate chronic pain symptoms? Conversely, can pain medications that modulate neuroinflammation slow down diseases like AD or PD if started early? Large-scale longitudinal studies are warranted to determine if effective chronic pain control in midlife leads to lower incidence of dementia or PD later on. Biomarker studies should further explore if pain biomarkers can predict neurodegenerative changes and vice versa.

The hypothesis connecting chronic pain to neurodegeneration via protein misfolding is supported by associative data and mechanistic plausibility, but direct causal proof in humans remains limited. Many studies are preclinical or cross-sectional. There is also heterogeneity in chronic pain (different etiologies may have different impacts on proteostasis) and in neurodegenerative diseases (not all AD patients have pain, etc.), so one size may not fit all. Additionally, interventions targeting misfolding in pain models are in early stages; translating these to patient care will require careful trials.

We need better animal models that develop both chronic pain and neurodegenerative pathology to test this bidirectional relationship. For example, a mouse model of chronic pain that is prone to Aβ accumulation could clarify if pain accelerates plaques. Omics studies on tissues from chronic pain patients might reveal a “proteostasis signature.” On the therapeutic front, early-phase trials of proteostasis-targeted drugs in chronic pain patients (especially those at high dementia risk) could be illuminating. Furthermore, the role of lifestyle interventions that improve proteostasis—exercise, diet, stress reduction—should be studied in the context of pain and cognitive health outcomes.

By focusing on protein misfolding and aggregation as a central mechanistic link between chronic pain and neurodegeneration, this review integrates perspectives from molecular neuroscience, clinical biomarker research, and experimental therapeutics into a unified framework. In contrast to prior literature that primarily addresses these conditions in isolation or as comorbidities, the current synthesis emphasizes proteostasis failure—including unfolded protein response activation, autophagy impairment, and prion-like propagation—as a shared and potentially modifiable pathway. Such an approach enables a deeper mechanistic understanding of their interplay and identifies novel therapeutic opportunities at the intersection of pain and neurodegeneration.

In conclusion, chronic pain and neurodegeneration appear to be linked by a web of common pathways, with protein misfolding and aggregation at the center of the web. Viewing chronic pain through the lens of proteostasis provides a novel understanding of its long-term effects on the nervous system. It suggests that treating pain is not only about symptom relief, but perhaps also about preserving the integrity of the brain and spinal cord. As research progresses, what we learn at this intersection could lead to innovative treatments that simultaneously quell pain and stave off neurodegeneration, significantly impacting public health as our population ages. The hypothesis that protein misfolding is a mechanistic bridge between chronic pain and neurodegeneration is a compelling one that merits further investigation—it challenges us to think of pain management as part of a broader strategy for neuroprotection and healthy aging.

## Figures and Tables

**Table 1:** Summary of the most relevant pharmacological agents and molecular targets involved in the modulation of protein misfolding, highlighting their mechanisms of action and potential therapeutic applications.

Author	Year	Drug/Target	Mechanism of Action	Preclinical/Clinical Evidence	Challenges and Future Directions
Jiang et al. [193]	2017	N-acetylcysteine	Restores glutathione, modulates HSPs, protects against proteasome inhibitor toxicity	Preclinical (neuroblastoma cell model)	Requires clinical trials in pain conditions
Gao et al. [194]	2019	Aspirin	Inhibits proteasomal degradation, promotes aggregate clearance via K63 ubiquitination	Preclinical (PD mouse model)	Repurposing needs validation; risk of systemic effects
Kan et al. [37]	2024	PKCε inhibitor	Reduces ER stress and autophagy activation	Preclinical (diabetic neuropathy model)	Needs human translation
Krukowski et al. [195]	2017	PFM-µ (Hsp70/Hsp90 inhibitor)	Inhibits chaperone activity and p53, prevents mitochondrial p53 aggregation	Preclinical (chemotherapy-induced neuropathy models)	No clinical trials
Stine et al. [196]	2019	Hsp90 inhibitors	Block opioid-induced analgesia via TLR4 modulation	Preclinical (mouse models of bone pain)	Mixed effects; may impair pain relief from opioids
Tikka et al. [197]	2001	Minocycline	Inhibits microglial activation and inflammatory cytokine production	Preclinical (primary neuronal cultures model)	Requires exploration in chronic pain with misfolding
Lisi et al. [198]	2012	Rapamycin (mTOR inhibitor)	Enhances autophagy, reduces inflammation	Preclinical (autoimmune encephalitis model)	Immunosuppression risk; unclear dosing for pain relief
Kraft et al. [199]	2021	Trehalose	Activates TFEB to enhance autophagy, clears protein aggregates	Preclinical (mice spared nerve injury model)	Sedation reported; require further safety and efficacy studies
